# Community pharmacies, drug stores, and antibiotic dispensing in Indonesia: a qualitative study

**DOI:** 10.1186/s12889-021-11885-4

**Published:** 2021-10-07

**Authors:** Astri Ferdiana, Marco Liverani, Mishal Khan, Luh Putu Lila Wulandari, Yusuf Ari Mashuri, Neha Batura, Tri Wibawa, Shunmay Yeung, Richard Day, Stephen Jan, Virginia Wiseman, Ari Probandari

**Affiliations:** 1 Center for Tropical Medicine, Faculty of Medicine, Public Health, and Nursing, Universitas Gadjah Mada, Yogyakarta, Indonesia; 2 Faculty of Medicine, Universitas Mataram, Mataram, Indonesia; 3 Department of Global Health and Development, London School of Hygiene & Tropical Medicine, London, UK; 4 School of Tropical Medicine and Global Health, Nagasaki University, Nagasaki, Japan; 5 Faculty of Public Health, Mahidol University, Bangkok, Thailand; 6 Aga Khan University, Karachi, Pakistan; 7 The Kirby Institute, University of New South Wales, Sydney, Australia; 8 Faculty of Medicine, Universitas Udayana, Bali, Indonesia; 9 Faculty of Medicine, Universitas Sebelas Maret, Surakarta, Indonesia; 10 Institute for Global Health, University College London, London, UK; 11 Department of Microbiology, Faculty of Medicine, Public Health, and Nursing, Universitas Gadjah Mada, Yogyakarta, Indonesia; 12 Department of Clinical Research, London School of Hygiene & Tropical Medicine, London, UK; 13 St Vincent’s Clinical School, University of New South Wales, Sydney, Australia; 14 The George Institute for Global Health, Sydney, Australia

**Keywords:** Indonesia, Antibiotics, Antimicrobial resistance, Community pharmacy, Pharmacist, Drug store

## Abstract

**Background:**

Inappropriate dispensing of antibiotics at community pharmacies is an important driver of antimicrobial resistance (AMR), particularly in low- and middle-income countries. Thus, a better understanding of dispensing practices is crucial to inform national, regional, and global responses to AMR. This requires careful examination of the interactions between vendors and clients, sensitive to the context in which these interactions take place.

**Methods:**

In 2019, we conducted a qualitative study to examine antibiotic dispensing practices and associated drivers in Indonesia, where self-medication with antibiotics purchased at community pharmacies and drug stores is widespread. Data collection involved 59 in-depth interviews with staff at pharmacies and drug stores (*n* = 31) and their clients (*n* = 28), conducted in an urban (Bekasi) and a semi-rural location (Tabalong) to capture different markets and different contexts of access to medicines. Interview transcripts were analysed using thematic content analysis.

**Results:**

A common dispensing pattern was the direct request of antibiotics by clients, who walked into pharmacies or drug stores and asked for antibiotics without prescription, either by their generic/brand name or by showing an empty package or sample. A less common pattern was recommendation to use antibiotics by the vendor after the patient presented with symptoms. Drivers of inappropriate antibiotic dispensing included poor knowledge of antibiotics and AMR, financial incentives to maximise medicine sales in an increasingly competitive market, the unintended effects of health policy reforms to make antibiotics and other essential medicines freely available to all, and weak regulatory enforcement.

**Conclusions:**

Inappropriate dispensing of antibiotics in community pharmacies and drug stores is the outcome of complex interactions between vendors and clients, shaped by wider and changing socio-economic processes. In Indonesia, as in many other LMICs with large and informal private sectors, concerted action should be taken to engage such providers in plans to reduce AMR. This would help avert unintended effects of market competition and adverse policy outcomes, as observed in this study.

## Introduction

Antimicrobial resistance (AMR) occurs when microbes evolve in response to the medicines that are used to treat them, reducing their therapeutic effectiveness. It is estimated that drug resistant infections will cause 10 million deaths annually by 2050 if appropriate measures are not taken [1]. In addition, infections caused by drug resistant microbes can lead to a two-fold increase in adverse outcomes in comparison with similar infections caused by susceptible strains. Adverse outcomes may be clinical (including death) or economic due to higher costs of care and indirect costs associated with loss of income [2, 3].

While AMR occurs naturally, it is accelerated by misuse and overuse of antimicrobials worldwide [4]. Global estimates indicate that 60% of antibiotics are sold without prescription in community pharmacies [5] and self-medication with antibiotics is a common practice, particularly in low- and middle-income countries (LMICs) [6]. Thus, a better understanding of these practices is crucial to inform national, regional, and global responses to AMR. In turn, this requires careful examination of the types of interactions between vendors and clients, sensitive to the context in which these interactions take place.

This paper examines these interactions within the country context of Indonesia. Surveys of antibiotic use in Indonesia have consistently reported inappropriate dispensing in the private sector amidst concerns about alarming rates of AMR [7, 8]. Notably, a study in Semarang province found that resistance of *Streptococcus pneumoniae* to cotrimoxazole was 45% and non-susceptibility rates to penicillin were around 24% [9]. *Escherichia coli* isolates from hospital discharged patients also showed high rates of resistance to ampicillin (73%), cotrimoxazole (56%) and ciprofloxacin (22%) [10].

In light of this, the government has taken several steps to tackle this problem, including the adoption of the first action plan on AMR in 2017 [11]. As in many other countries, however, AMR policy and interventions in Indonesia have largely focused on the public health system with little attention to the private sector [12]. In addition, the evidence to inform policy has been derived primarily from prevalence or knowledge, attitude and practice surveys [13, 14], with inadequate understanding of contextual drivers of AMR and associated practices. Considering this gap in knowledge, our paper presents findings from a qualitative study that aimed to better understand how and why non-prescribed antibiotics are dispensed at community pharmacies and drug stores in Indonesia. After a description of the research context and methods, the following sections report the study findings and discuss their implications for AMR policy in Indonesia and similar contexts.

## Private drug sellers and AMR in Indonesia

﻿Indonesia is the fourth most populous country in the world, with a population of more than 270 million [15]. Following decades of economic growth, the country recently advanced to upper-middle income status in its World Bank classification. Health care is delivered by a mixed public-private health system [16]. The public health sector is decentralised and consists of community health centers (*puskesmas*) at the subdistrict level and state hospitals at the district and provincial levels. The private sector is large and diverse. It includes not-for-profit faith-based organisations, for-profit private practices, clinics and hospitals, and practitioners of traditional medicine. In 2014, the government introduced a new health insurance scheme – the *Jaminan Kesehatan Nasional* (JKN) - to increase access to care and move towards universal health coverage. Under this scheme, Indonesian citizens who pay the monthly premium (or are eligible for an exemption) are entitled to receving care and essential medicines at public health facilities and participating private providers, including community pharmacies [17].

Pharmacies and other private drug sellers are generally the first port of call for around 90% of patients with mild illness [18]. This is a prolific and dynamic business sector which includes big retail chains, independent pharmacies (*apotek*), drug stores (*toko obat*) and unlicensed grocery stores (*warung*) that also sell over-the-counter drugs such as common cold medicines, antipyretics, and analgesics. The expansion of this sector can be traced back to the 1970s when President Suharto’s government embraced economic liberalisation, opening the country to foreign investments. In this period, more than 30 international pharmaceutical companies established operations in Indonesia, and generic products began to be manufactured locally [19]. In the 2010s, the government implemented several pricing policies to make generic drugs more affordable, promoting further expansion of the pharmaceutical sector [20]. Today, it is estimated that more than 22,000 pharmacies and 5000 licensed drug stores operate throughout the country [21].

By Indonesian law, antibiotics can only be sold by a pharmacist after presentation of a prescription and drug stores are not allowed to sell antibiotics [22]. In addition, a qualified pharmacist must always be available at pharmacies during opening times, although ownership of a pharmacy is not restricted to pharmacists [23]. Despite these regulations, past studies [7, 14] and a recent survey [8] found that inappropriate antibiotic dispensing is widespread among community pharmacies and drug stores, raising concerns about their role in promoting AMR.

In order to address these problems, in 2017 the government adopted a national action plan to combat AMR in line with the WHO global guidelines. Among other provisions, this plan aims to improve awareness and knowledge of AMR in the general public and among health professionals (including pharmacists), and to “strengthen the enforcement of regulations on antimicrobial post-marketing surveillance” [24]. Since 2015, the Ministry of Health has also implemented a community-based program to promote responsible self-medication, called the “Community Smart Use of Medication Movement” (*Gerakan Masyarakat Cerdas Menggunakan Obat*), known as GeMa CerMat, where pharmacists are trained to provide information on medicines to their communities. Despite some progress, the implementation of these initiatives has been slow and private sector engagement remains a significant policy challenge [25].

## Methods

### Research design and settings

The qualitative study presented here was part of a larger study of AMR in Indonesia conducted in 2019, which found high rates of inappropriate dispensing and poor consultation practices at pharmacies and other drug stores [8]. In the same study locations, we conducted interviews to better understand the ‘how’ and ‘why’ of non-prescribed antibiotic dispensing. In keeping with the principles of phenomenology [26], the qualitative study focused on individual experiences and perceptions of the social actors involved in the final stage of antibiotic dispensing – those who sell antibiotics and their clients. Data collection involved in-depth interviews, conducted in an urban location (Bekasi) and a semi-rural location (Tabalong) to capture different markets and contexts of access to medicines and different densities of pharmacies in these locations. Bekasi is an industrial area located in West Java Province and a satellite city of the national capital Jakarta. It has a population of about 3.5 million, largely consisting of internal migrants and commuters from other parts of Java. Tabalong is a district located in South Kalimantan Province with a population of 250,000 [27]. In 2018, the number of pharmacies in Bekasi and Tabalong was 534 and 31 respectively, while the number of drug stores was 87 and 31 respectively [21].

### Study participants and data collection

In both study settings, a list of all registered pharmacies and drug stores (collectively defined here as “drug retail outlets”, DRO) was obtained from the district health offices and sorted by subdistrict. In each subdistrict, pharmacies and/or drug stores were randomly selected from the registry and telephoned to ask if they were willing to take part in the study and to schedule a time to conduct an interview. In each pharmacy and drug store, we aimed to recruit two categories of respondents: (1) vendors responsible for drug dispensing, including pharmacists, shop attendants, and owners); and (2) adult clients, who were approached and invited to participate in the interview after they had completed their interaction with the vendor.

All interviews were conducted face-to-face in Indonesian (Bahasa Indonesia) by the first author and other Indonesian researchers with training in social research methods using a semi-structured topic guide, tailored to each category of participants. Most interviews with vendors focused on: the type of business; supply and sales of antibiotics; antibiotic dispensing practices; awareness of antibiotic resistance; and awareness of regulations and compliance. Interviews with clients mainly focused on health seeking behaviour and their use and knowledge of antibiotics. After each interview, summary notes were taken to refine the interview guidelines for the following interviews and to determine saturation. All interviews were audio-recorded and lasted between 25 and 70 min. The interviews were conducted in August and September 2019.

### Data management and analysis

The interviews were transcribed verbatim and processed to generate an initial set of codes after iterative, inductive reading of the transcripts. The coding scheme was subsequently reviewed and refined after discussion with the study team to reduce subjective biases. The analysis was informed by two main concerns: to understand the types of interactions leading to inappropriate dispensing of antibiotics; and to explore factors shaping these interactions and their outcomes. Thematic content analysis and coding were performed using NVivo version 9.

## Results

### Overview

In total, interviews were conducted at 21 pharmacies and 10 drug stores. As detailed in Table 1, 31 interviews were conducted with vendors (owners, pharmacists, and attendants) and 28 with adult clients in the 19–65 age range. In Bekasi, pharmacies and drug stores of different types and size were clustered near clinics or residential complexes or lined along the main city roads. In Tabalong, most pharmacies were located near the district centre although a few drug stores were in peripheral areas. The majority of DROs were small independent businesses serving about 15 to 20 customers every day; only a few pharmacies in Bekasi were part of retail chains. By Indonesian law, pharmacies and drug stores must supply medicines, including antibiotics, from wholesale distributors. However, a few pharmacists admitted they would regularly restock at large retail pharmacies since these would offer better deals and, unlike wholesalers, would not apply minimum order requirements.

**Table 1:** Study sample

	Bekasi	Tabalong	Total
**Pharmacies**			
Owner	3	2	5
Pharmacist	2	1	3
Owner and pharmacist	7	5	12
Pharmacy attendant	2	–	2
Client	13	11	24
**Drug stores**			
Owner	1	6	7
Drug store attendant	1	1	2
Client	2	2	4
**Total**	**31**	**28**	**59**

Pharmacists in both locations explained that people often medicate with drugs purchased at their stores because they have little money to pay for a private doctor or little time to visit a public health centre, where they often have to wait in long queues:

“Well frankly… if they go to the doctor, they will be charged 75,000 or 100,000 rupiah [US$ 5-7] for the consultation. That’s quite a lot. If they come to my place, they can get some information about how the medicine works, and they don’t have to pay that much… that’s why they like it.” (Male pharmacist, Bekasi, V05B).

Similarly, clients said that pharmacies and drug stores are more accessible than public health facilities and open in the evening:

“It takes time to go to the primary health centre. It’s far, then we have to wait. I only visit the health centre if I feel really sick.” (Male client, 29, Tabalong, C03T)

“I don’t have time to go to the health centre in the morning (…) I must work.” (Female client, 54, Bekasi, C05B)

In this context, antibiotics could be easily purchased without prescription at both pharmacies and drug stores:

“*Is it easy to get antibiotics?* Well… you can find them everywhere.” (Male client, 33, Bekasi, C02B)

“Maybe 10% of my sales are antibiotics, and the proportions of prescribed and non-prescribed antibiotics is about 30 and 70%.” (Female pharmacist, Bekasi, V012B)

“Drug stores are not supposed to keep antibiotics, but they often sell ampicillin and amoxicillin. Sometimes they also sell ciprofloxacin and cefadroxil although this is less common.” (Male owner/pharmacist, Tabalong, V01T)

“It is so easy to get [unprescribed] antibiotics here… sometimes even the small vendors around here have them.” (Male owner/pharmacist, Tabalong, V01T)

In the following sections, in line with the study objectives, we will describe the interactions leading to inappropriate antibiotic dispensing and their drivers.

### Antibiotic dispensing patterns

A common pattern of non-prescribed antibiotic dispensing was the direct request of antibiotics by clients as part of a self-medication strategy, based on previous experiences in the public sector or advice from relatives or friends. As reported by vendors, many clients walked into their pharmacy or drug store and ordered a specific antibiotic either by giving a generic/brand name or showing a used package:

“Not many people ask for advice, they usually come to buy specific drugs (…) When they want to buy antibiotics, they usually ask for amoxicillin.” (Female pharmacy attendant, Bekasi, V014B)

“Many customers were prescribed antibiotics from the primary health centre and then they buy [the same antibiotic] from us. They remember the name and mention it to us.” (Male pharmacist, Bekasi, V05B)

“They come into my shop and say what they want to buy, like ‘I want amoxicillin and paratusin [a cough and cold medicine]’.” (Female pharmacist, Bekasi, V013B).

These accounts were mirrored in interviews with customers:

“*How did you first get the antibiotics?* From the physician at the primary health centre, I was sick and given prescription. *A long time ago?* Yes. *Did you buy the same medicine again?* Yes, but I no longer visit the health centre for consultations.” (Male client, 39, Tabalong, C05T)

“Some time ago I got the medicine with the doctor’s prescription. And then it was finished, so I bought it again (…) I usually bring the used package, or I tell the name.” (Female client, 39, Bekasi, C07B)

“I bought amoxicillin at the drug store, with no prescription. I brought sample package from my neighbour – I was told that I can get the medicine at the drug store or pharmacy.” (Female client, 47 Bekasi, C015B)

A less common pattern involved pharmacists recommending an antibiotic, often based on a brief assessment of the patient:

“I will suggest antibiotics to those who look sick, for example those with toothache or respiratory infection…” (Female pharmacist, Bekasi, V012B)

“I recommend antibiotics only if there are signs of infection.” (Female pharmacist, Tabalong, V03T)

Lastly, some pharmacists said that they would refer clients with severe symptoms to health care facilities, especially when the patient was a child:

*“*If the patient is really unwell, I would recommend them to go to the hospital, for example if a child has fever for more than 3 days.” (Male pharmacist, Tabalong, V012T).

### Drivers of inappropriate antibiotic dispensing

In addition to the interactions between clients and vendors concerning the sale of antibiotics, the interviews sought to explore factors that shaped these interactions and their outcomes. These factors are described in turn below.

#### Misconceptions about antibiotics

Lack of knowledge or misinformation about antibiotics were common in the study locations. Many vendors had no training in pharmacy and were not aware of AMR. In addition, pharmacists in both Bekasi and Tabalong explained that their customers demanded antibiotics indiscriminately for any condition:

“There are many clients asking for antibiotics, maybe 30-40%. They want antibiotics for everything… even for muscle pain…I don’t know what’s wrong. Sometimes they are very stubborn… if we try to explain that antibiotics should be used for other [diseases], they often insist… they still want them.” (Female owner/pharmacist, Bekasi, V010B)

“Most people here believe that all diseases can be treated with antibiotics.” (Male drug store owner, Tabalong, V011T)

In keeping with these comments, many clients believed that antibiotics are “powerful” (*manjur*) medicines that can be used to effectively treat a common cold, inflammation, toothache, cough, and even as an energy booster:

“[Antibiotics] reduce fever faster… they improve our antibodies.” (Female client, 28, Tabalong, C08T)

“When I have a cough, I usually take amoxicillin, paracetamol and konidin [cold medicine]. If I only take konidin, I don’t feel any effect. But when I mix it with amoxicillin and paracetamol, I immediately get better.” (Male client, 65, Tabalong, C012T)

“*Why did you take amoxicillin?* I often feel tired and sleepy… I am a driver, so it is dangerous if I’m sleepy.” (Male client, 39, Tabalong, C05T)

A few clients stated they felt ignorant or lacked knowledge about antibiotics and therefore were reluctant to use them without expert advice. For example, a customer in Bekasi said:

“I never buy such drugs [antibiotics] because I don’t really know. If I don’t have the prescription, I don’t want to take them.” (Male client, 60, Bekasi, C01B).

As the next citations illustrate, poor knowledge of antibiotics was often associated with different forms of malpractice such as the storage of leftover antibiotics at home for future use.

“*What kind of medicine do you keep at home?* Amoxicillin, paracetamol… *How frequently do you buy these medicines?* I buy them when they run out, sometimes every week. *Do you use them for all family members?* Yes.” (Male client, 22, Bekasi, C03B).

*“When was the last time you were sick?* I think 3-4 months ago. *What did you have?* Nothing special…just fever and headache. *What did you do?* I immediately took antibiotics. *Where did you get them?* I had some at home.” (Male client, 33, Bekasi, C02B)

“We have medicine stock at home… When somebody is sick, I give paracetamol and amoxicillin. That’s all.” (Female client, 48, Tabalong, C07T)

#### Market competition and the unintended consequences of health policy reform

The struggle to survive in an increasingly competitive market was another driver of inappropriate antibiotic dispensing. Some pharmacists reported a daily revenue of IDR 300,000–500,000 (USD 20–30) and a net profit of only IDR 50,000–100,000 (US$ 3–7) due to high operational costs and competition. According to some participants, the financial viability of some pharmacies had been negatively affected by the introduction of free medicines under the JKN. Pharmacists who were not part of this scheme complained they had experienced a decline in clients and income, and were therefore more likely to use coping strategies such as the dispensing of non-prescribed medicines:

“I can say that pharmacies are no longer profitable, because of the JKN. So don’t be surprised if many pharmacies are going bankrupt.” (Male pharmacist/owner, Tabalong, V02T)

“It has been very difficult for three years. We have high operational costs and we make little profit… I feel like I am volunteering… I am working only for personal satisfaction - what we earn is not much.” (Female pharmacist, Tabalong, V03T)

“It’s getting hard… you know… If we deny antibiotics, they can buy them with no prescription at other places… and our income will decrease.” (Female pharmacist, Bekasi, V08B)

In addition, due to financial constraints, some owners of pharmacies admitted they could no longer employ a full-time pharmacist. In Tabalong, this problem was compounded by challenges to attract and retain qualified pharmacists as these would prefer to work in the cities rather than in rural areas. As a result, pharmacies in both locations were often staffed by unqualified attendants, with little knowledge of antibiotics and regulations governing their use.

#### Limited decision-making power of pharmacists who do not own the pharmacy

Other pharmacists explained that inappropriate dispensing could occur even when a qualified pharmacist was available. One pharmacist in Bekasi said that this was often a result of the power imbalance between the pharmacist and the pharmacy owner, since the latter had the “highest level of decision-making”, and therefore could influence dispensing practices even when these were not in line with the regulations. Similarly, another pharmacist said she could not deny antibiotics without prescription because she had no power (Female pharmacist/owner, Bekasi, V010B).

#### Weak enforcement of regulations

There was widespread consensus that the enforcement of regulations was weak, despite the active role of professional pharmacist associations in both study locations. In Bekasi, one drug store owner said she could sell antibiotics easily due to lack of supervision:

“*Did you receive any visits from the district health office recently?* Never. *So you can sell antibiotics easily?* Yes… I can sell drugs like amoxicillin. *Why amoxicillin?* Because it is a common drug everywhere.” (Female drug store owner, Bekasi, V019B)

This narrative was echoed in the views of some pharmacists, who confirmed that dispensing practices at both pharmacies and drug stores were not really monitored:

“There should be strict monitoring (…). Drug stores are not supposed to sell antibiotics, but they often have ampicillin and amoxicillin. Sometimes they also sell cipro[floxacin] and cefadroxil although this is less common.” (Male pharmacist, Tabalong, V01T)

## Discussion

**Fig. 1:**
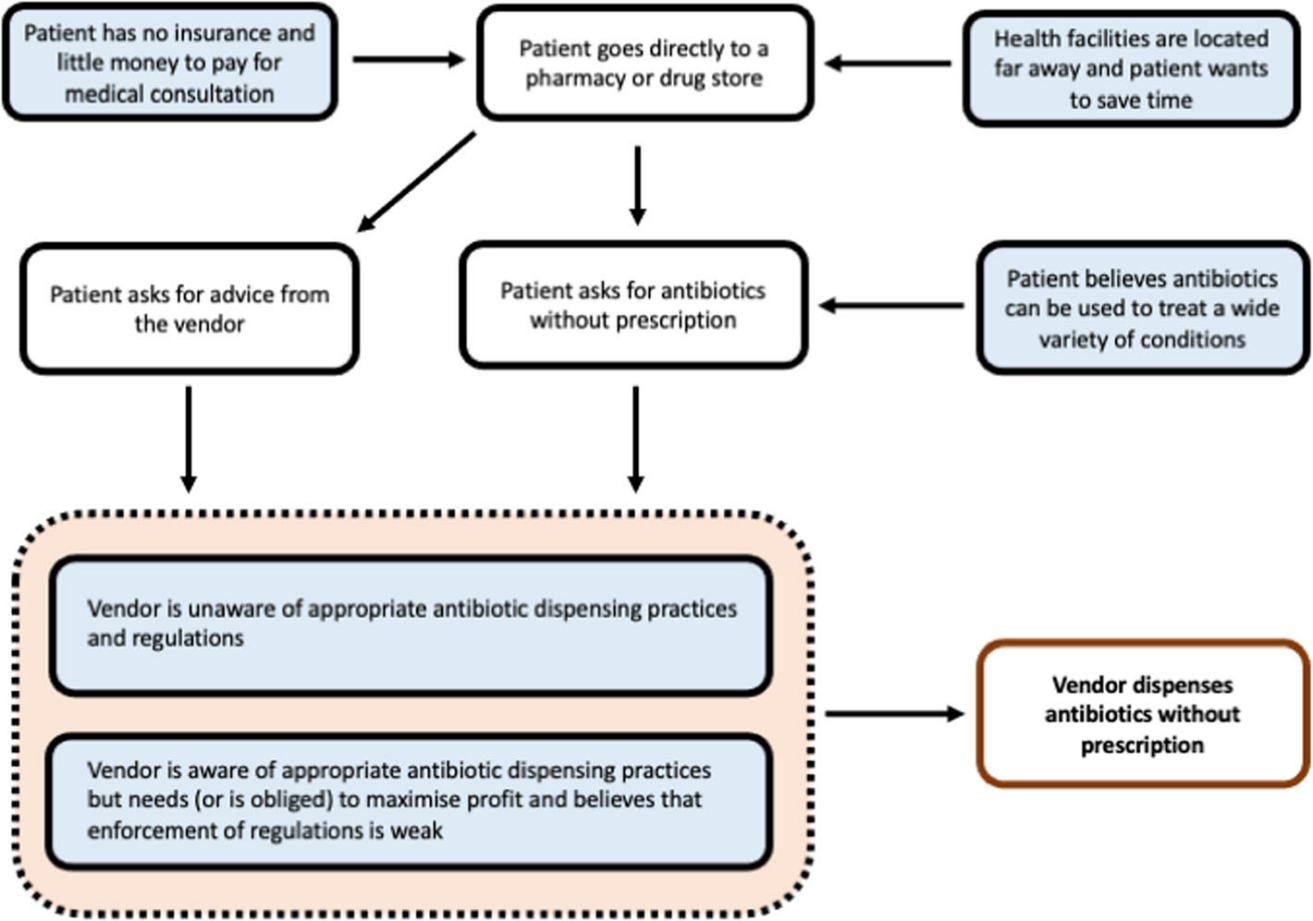
Diagram illustrating pathways leading to inappropriate antibiotics dispensing from the perspective of patients and vendors. Boxes in blue are cognitive processes or states. Boxes in white are actions

This study sought to gain a better understanding of antibiotic dispensing practices at community pharmacies and other drug stores in Indonesia. As documented in the interviews, participants perceived that self-medication with drugs purchased at DROs provides a convenient and low-cost strategy to relieve symptoms. Further, we identified two types of interactions or scenarios resulting in inappropriate dispensing. In the first type, clients directly requested antibiotics without prescription, either by stating their generic/brand name or by showing an empty package or sample. In the second scenario, which we found to be less common, vendors “prescribed” antibiotics to clients who presented with symptoms and asked for advice, often based on a brief assessment (Fig. 1). As found in other studies, these two patterns highlight the role of both direct and indirect self-medication practices in driving inappropriate antibiotic use [28, 29].

Our findings also cast light on the different drivers of inappropriate antibiotic dispensing in the study locations. On the demand side, similar to recent findings in China [30] and Thailand [31], many participants in our study believed that antibiotics are multi-purpose anti-inflammatory drugs which can be used to treat almost any condition. In general, self-medication with antibiotics was seen as an appropriate, affordable and convenient practice to relieve symptoms, while concerns about long waiting times and costs discouraged the utilisation of public health facilities as found in other studies [32]. On the supply side, economic challenges and market competition, combined with widespread availability of cheap antibiotics and lack of adequate information on AMR, induced pharmacies to sell non-prescribed antibiotics. While some of these issues have been highlighted in previous work in Indonesia [18, 33] and elsewhere in Asia [34–38], a key finding in our study is that policy reforms to improve access to affordable services and medicines may have unintended and undesirable effects on pharmacy practice and, ultimately, the control of AMR. Since the introduction of the JKN in 2014, a substantial increase in the utilisation of public health centres has been reported [39]. In contrast, independent pharmacies outside the JKN network face new challenges as many of their former clients choose to receive subsidised care (and medicines) in the public sector. As documented in some interviews, this may induce coping strategies to minimise economic loss such as the inappropriate dispensing of antibiotics. Thus, our study highlights the need for stronger private sector engagement in policies to promote UHC and appropriate antibiotic use, especially in countries such as Indonesia where the private sector is such a popular source of care.

Lastly, our study highlights shortcomings in the regulatory enforcement and monitoring of drug sales, particularly in relation to human resources. As discussed, one important gap is that in many places qualified staff are not always in attendance – a trend that is well known in Indonesia as in other parts of Asia [34, 40]. This is a complex problem which can be associated with multiple factors including weak supervision from local authorities, challenges in attracting and retaining pharmacy professionals in rural areas, and inadequate salaries. However, in keeping with findings from India [41, 42], our study also found that attendance by pharmacists does not guarantee appropriate dispensing, partly due to potential conflicts of interest between the professional ethics of pharmacists and the economic incentives of those who own and manage the pharmacies. Another reason is that some pharmacists are reluctant to deny antibiotics to clients who ask for them, in fear that they will take their business elsewhere. This suggests that the educational curriculum for pharmacists should be reformed to include specific training on interpersonal communication skills to manage patient demand for medications [43, 44].

In sum, the findings from this study highlight the multi-faceted nature of AMR and the complex mix of drivers of antibiotic dispensing in the community, inviting some reflections on policy implications. In the communities, recent evidence indicates that interventions to educate pharmacists alone may be ineffective unless these are supported by campaigns on proper antibiotic use in the general population [45]. Given that community members are often introduced to the use of antibiotics in the public sector, doctors and nurses at public health centres could be involved in educational campaigns, providing more information on AMR when prescribing. Yet it is well recognised that interventions solely focused on individual behaviour are unlikely to succeed, given the key role of wider contextual drivers [46]. Indeed, past experiences indicate that multi-faceted interventions are more effective than single interventions to reduce inappropriate antibiotic use. For example, positive outcomes have been achieved in other LMICs where stronger regulatory enforcement was combined with educational programmes such as training of dispensers and public awareness campaigns [47–50]. Available evidence also suggests that interventions targeted to different stakeholders (including doctors, nurses, pharmacists, patients, and the public) are more likely to be effective than those that only focus on one group [51]. On the supply side, incentive systems could also be devised such as renewing licenses only to those who comply with the regulations and appropriately dispense medicines. In pharmacies, this could also be a requirement for inclusion in public health schemes such as the JKN network.

### Study limitations

The findings presented here offer qualitative insights into antibiotic dispensing practices and their determinants in the study locations but cannot be generalised to the whole country and beyond. In addition, all interviews were conducted inside the pharmacies and drug stores; in these environments, participants (both clients and vendors) may have felt some pressure to provide socially desirable answers and perhaps understate the extent and willingness to engage in unauthorised practices. In addition, this study focused on behaviour and practices in the communities. Therefore, it did not include other important categories of stakeholders influencing antibiotic dispensing such as policy makers, implementers of programs to control AMR, and representatives of professional associations and business interests in the pharmaceutical sector. Lastly, a larger number of drug stores in the study sample might have provided additional insights, so we cannot be sure saturation was achieved for this type of outlets.

## Conclusion

Findings from this study reveal that inappropriate dispensing of antibiotics is the outcome of a complex set of interactions between vendors and clients, shaped by wider and changing socio-economic processes. As such, it contributes knowledge and insights that can be used to refine existing models and conceptual frameworks of behavioural patterns and their determinants leading to inappropriate dispensing [42]. In Indonesia, as in many other LMICs with large and informal private sectors, concerted action should be taken to engage both pharmacies and drug stores in plans to reduce AMR. This would help avert unintended effects of market competition and adverse policy outcomes as observed in this study.

## Data Availability

The anonymised dataset is available upon reasonable request.

## Abbreviations

AMR: Antimicrobial resistance
JKN: Jaminan Kesehatan Nasional (National Health Insurance)
LMICs: Low- and Middle-Income Countries
DRO: Drug Retail Outlet
UHC: Universal Health Coverage
WHO: World Health Organization
